# All-Trans Retinoic Acid Modulates ORMDL3 Expression via Transcriptional Regulation

**DOI:** 10.1371/journal.pone.0077304

**Published:** 2013-10-29

**Authors:** Li-Li Zhuang, Bo-Xian Huang, Jie Feng, Liang-Hua Zhu, Rui Jin, Ling-Zhi Qiu, Guo-Ping Zhou

**Affiliations:** 1 Department of Pediatrics, The First Affiliated Hospital, Nanjing Medical University, Nanjing, Jiangsu Province, China; 2 School of Life Science and Technology, China Pharmaceutical University, Nanjing, Jiangsu Province, China; 3 State Key Laboratory of Reproductive Medicine, Clinical Center of Reproductive Medicine, The First Affiliated Hospital, Nanjing Medical University, Nanjing, Jiangsu Province, China; Baylor College of Medicine, United States of America

## Abstract

All-trans retinoic acid (ATRA) is an active metabolite of Vitamin A, it shows protective effects on asthma, including maintains airway epithelial integrity, inhibits asthma effector cells differentiation, modulates immune response, et al. However, the promoting effect of ATRA on Th2 response has restricted the clinical application of ATRA in asthma treatment. ORMDL3 is a candidate gene of childhood onset asthma, and high-transcript of ORMDL3 is associated with the development of asthma. Here we show that ATRA increases ORMDL3 production in vitro via inducing PKA-dependent CREB phosphorylation which in turn binds to the CRE element in promoter region of ORMDL3 and initiates ORMDL3 transcription. This finding is in consistent with the previous reports that ATRA could regulate target genes without the presence of retinoic acid response element (RARE) in promoter region but through other signals such as PKA/CREB. Nevertheless, in the present study, the traditional signal pathway of ATRA, retinoic acid receptor (RAR) signal transduction pathway, indirectly modulated ORMDL3 expression. RAR-α agonist (Am-80) increased ORMDL3 production even though there was no RARE in ORMDL3 promoter, introns or 3′-downstream region. Besides, the signal of RAR might differ from that of ATRA since Am-80 failed to induce CREB activation. In conclusion, our data indicate that ATRA facilitates ORMDL3 production probable through PKA/CREB, and this may be a starting point for more detailed mechanism researches on ATRA and asthma.

## Introduction

Asthma is a complex trait characterized by airflow obstruction, airway inflammation, and persistent airway hyperreactivity. It is caused by a combination of genetic predisposition and environmental influences. So far, more than 150 genes have been associated with asthma and related phenotypes. ORMDL3 was identified as an asthma candidate gene by Genome-wide association studies in diverse human populations [1], [2], [3], [4], [5]. Increased transcript level of ORMDL3 had been found in Epstein-Barr-virus-transformed lymphoblastoid cell lines from children with asthma [1] and in human rhinovirus stimulated peripheral-blood mononuclear cells [6]. ORMDL3 mRNA contents in the peripheral blood of recurrent wheeze patients [7] and in bronchial epithelial cells of asthmatic subjects were higher compared to controls [8]. Besides, ORMDL3 production of airway epithelial cells of mice could be induced by allergen sensitization [9]. These studies indicated that high expression of ORMDL3 might associated with the development of asthma, but the mechanism of ORMDL3 to the pathogenesis of asthma is poorly understood. Functional studies indicated that ORMDL3 is involved in the processes of endoplasmic reticulum stress induction [10], sphingolipid regulation [11] and T-lymphocyte activation [12].

At present, the combination of inhaled glucocorticoids and β_2_-adrenoceptor agonists is the major treatment for asthma. Despite most patients have a response to this combination therapy, it is estimated that as many as 20% of patients remain poorly controlled [13]. Optimization of the current treatment options is very urgent.

Vitamin A plays important roles during embryonic development, cell growth and differentiation, immune responses, et al. In respiratory system, Vitamin A is involved in lung morphogenesis [14], airway epithelium phenotype maintenance [15], airway autonomic nervous regulation [16] and immune responses [17]. Epidemiologic studies showed that serum Vitamin A was lower in children with asthma compared with controls, and Vitamin A deficiency might be associated with the development of asthma and allergic disorders [18]. Thus, Vitamin A is supposed to be of therapeutic value for asthma.

All-trans retinoic acid (ATRA), the major metabolite of Vitamin A, regulates gene transcription mainly through two families of nuclear receptors, the retinoic acid receptor (RAR-α,-β,-γ) and the retinoid X receptor (RXR-α,-β,-γ) [19]. ATRA modulates several biological functions of airway epithelial cells, eosinophils, and immune cells via RAR/RXR signals. Pharmacological concentration of ATRA inhibits the differentiation, mature and function of the main effector cell populations of allergic airway inflammation and allergic bronchial asthma, including eosinophil cells [20], basophile cells [21] and mast cells [22], [23]. ATRA maintains the function of airway cholinergic receptors and alleviates airway hyperresponsiveness [24]. In addition, ATRA suppresses signaling of phosphatidylinositol 3 kinase (PI3K)/Akt and blocks platelet-derived growth factor-induced actin reorganization and airway smooth muscle cells migration, suggesting that ATRA may be of inhibitory effect on airway remodeling [25].

We previously reported that cAMP/PKA/CREB signaling plays an important role in ORMDL3 regulation [26]. Prior studies illustrated that cAMP/PKA pathway is an integrated part of ATRA signal network [27]. ATRA stimulates target genes expression though inducing CREB phosphorylation, which in turn initiates transcription [28], [29]. Here, we demonstrate that ATRA up-regulates ORMDL3 expression via transcriptional regulation probably by PKA/CREB pathway in mouse embryonic fibroblast cells NIH3T3, human bronchial epithelial cells BEAS-2B and human type II alveolar lung epithelium cells A549.

## Materials and Methods

### Cell Preparation

NIH3T3 and A549 cells were obtained from the American Culture Collection (ATCC). BEAS-2B cells were kindly provided by Dr. Hongwei Wang (The medical college, Nanjing University, China) who purchased it form ATCC. Cells were cultured in Dulbecco's modified Eagle's medium (DMEM) supplemented with 10% heat inactivated fetal bovine serum, penicillin (100 unit/ml) and streptomycin (100 μg/ml). Cells were maintained in an incubator at 37°C and equilibrated with 5% CO_2_ and subcultured using standard cell culture techniques.

### Reagents and Medications

ATRA, Forskolin, H-89 and Am80 (Sigma-Aldrich, St. Louis, MO) stock solutions were made in DMSO, and added directly to the cell culture media to produce the final concentration.

### Plasmids, Transfection and RNAi

The mouse ORMDL3 genomic DNA fragment −74/+141 (the transcriptional start site (TSS) was designed as +1), which was the functional proximal minimal promoter and contained a CREB-binding site, was inserted into pGL3-basic vector (Promega) between *Kpn* I and *Bgl* II restriction sites in the poly linker upstream of the luciferase reporter gene, named as pGL3-74 as previously described by us [26]. Internal CREB-binding site deletion mutation construct (del-CRE) was performed as previously described [26]. The CREB expression plasmid Y/F-CREB was kindly provided by Dr. M. Montminy (The Salk Institute, CA, USA), and the corresponding control plasmid pcDNA3 was purchased from Invitrogen (Carlsbad, CA, USA). For RNAi assay, three individual oligonucleotides specific for CREB were used and the sequences are following (sense): 5′-GAUUCACAGGAGUCUGUGGtt-3′, 5′-UACAGCUGGCUAACAAUGGtt-3′ and 5′-CCAAGUUGUUGUUCAAGCUtt-3′; and the control siRNA is: 5′-UUCUCCGAACGUGUCACGUtt-3′. All siRNAs were synthesized and high-performance purified (GenePharma). Plasmids and siRNA were transfected using Lipofectamine™ 2000 (Invitrogen) and the effectiveness of overexpression or interference were examined as previously described [26].

### Dual-Luciferase Reporter Assays

Cells were seeded into 96-well plates (15,000 cells/well) and grown overnight in phenol red-free DMEM supplemented with 10% of charcoal-stripped FBS. CREB expression plasmid (100 ng) or empty vector (pcDNA3) was individually together with pGL3-74 (100 ng) and co-transfected into cells using Lipofectamine™ 2000. For RNAi assay, CREB siRNA or control siRNA was individually with pGL3-74 and co-transfected into cells. Cells were treated with 1 μM ATRA 24 h after transfection and further cultured for another 24 h before luciferase assays carried out. The pRL-TK plasmid (Promega, 2 ng/sample) containing the Renilla luciferase gene driven by the herpes simplex virus thymidine kinase promoter was co-transfected with the constructs, and the luciferase activity was normalized. The preparation of cell lysates and measurement of luciferase activities were performed using the Dual Reporter Assay System (Promega) and TD-20/20 Turner Designs luminometer.

### RNA Extraction and Quantitative Real-Time PCR

Total RNA was extracted using the Trizol Reagent (Invitrogen) and subsequently reverse transcribed using the PrimeScript RT Master Mix Perfect Real Time kit (TaKaRa). Quantitative Real-Time PCR was performed on the Step One Plus Quantitative Real-Time PCR System (Applied Bio-systems) using SYBR Green (TaKaRa). The PCR cycling conditions were: 40 cycles of 30 s at 95°C, 1 min at 60°C. Fold-induction was calculated using the formula 2^−(ΔΔCt)^. PCR product quality was monitored using post-PCR melt curve analysis. The specific primer pairs were below: ORMDL3: sense: 5′- CCCTGTGGGTTTGAACTCCTG-3 antisense: 5′-GTCGAAGTGAACCCGCTTCTG-3′; β-actin: sense: 5′-AACAGTCCGCCTAGAAGCAC-3′; antisense: 5′-CGTTGACATCCGTAAAGACC-3′.

### Western Blot

Protein of cells were extracted using a Total Protein Extraction Kit (Keygentec, China) and protein concentration were determined using the Bio-Rad Protein Assay (Bio-Rad). Protein were run on 12% SDS-PAGE gels, blotted on nitrocellulose membrane, and immunodetected with primary antibodies against P-CREB (Santa Cruz), CREB (Santa Cruz) and ORMDL3 (Abcam). β-Tubulin (Santa Cruz) was detected as loading control. Chemoluminescence signals were quantified using an ECL imager, and analyzed using Quantity One software (Bio-Rad).

### Statistical Analysis

All data were presented as mean ± SE and were analyzed using one-way analysis of variance and Tukey's comparison test, using SPSS Software (version 16.0). Data were representative of at least three independent experiments. *P*<0.05 was considered significant.

## Results

### Effects of ATRA on CREB Activation in NIH3T3 cells

**Figure 1:**
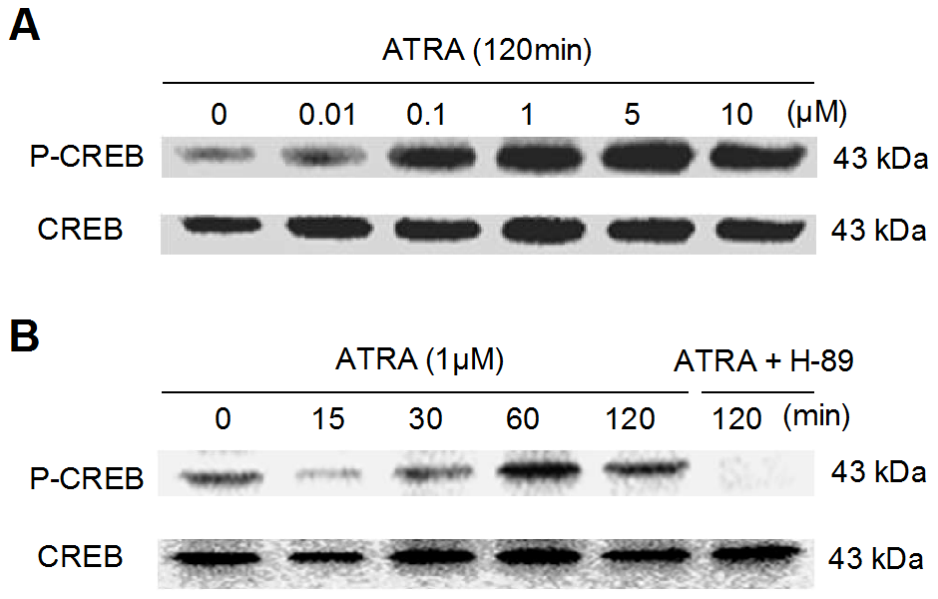
ATRA induced CREB phosphorylation in a time- and dose-dependent manner. (A and B) ATRA induced CREB activation in a time- and dose-dependent manner in NIH3T3 cells. (B) Pre-incubation with H-89 (15 μM) for 1 h blocked ATRA-induced CREB activation.

Induction of CREB phosphorylation by ATRA were analyzed by Western blot. As detailed in Fig. 1A, ATRA induced CREB activation in a dose-dependent manner. CREB was activated in the presence of as little as 10 nM ATRA, and the strongest activation was achieved at 5 μM. Since the therapeutic level of ATRA in human plasma is about 1–2 μM [25], we selected the concentration of 1 μM for the time-course examination. As seen from Fig. 1B, elevated phosphorylation level of CREB could be detected after incubated with ATRA for 30 minutes, and could last to 120 minutes.

### Induction of CRE-dependent ORMDL3 Transcriptional Activity by ATRA

**Figure 2:**
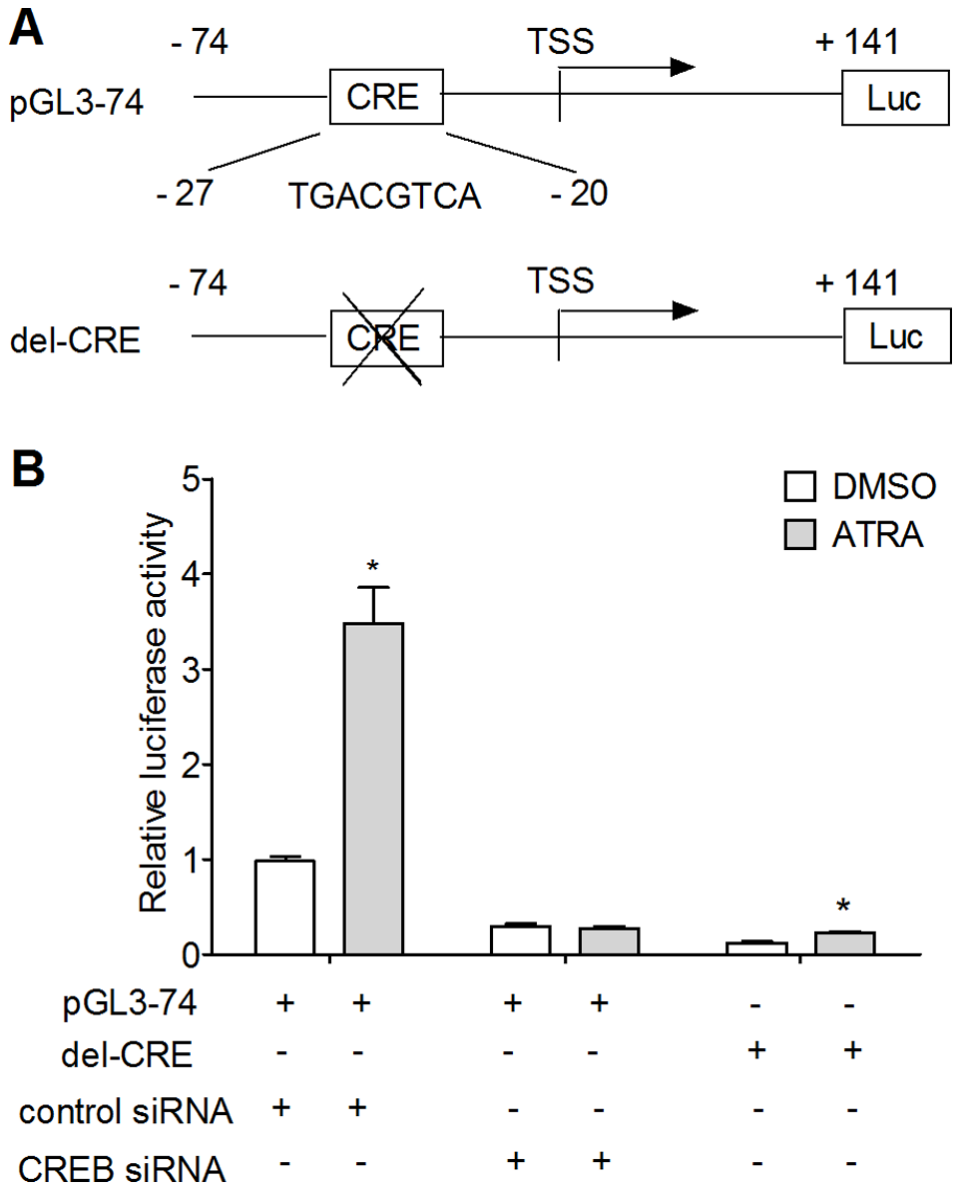
ATRA up-regulated CRE-dependent mouse ORMDL3 promoter transcriptional activity in NIH3T3 cells. (A) Schematic diagram of the mouse ORMDL3 gene core promoter-driven luciferase plasmid pGL3-74 and CRE-element deletion mutation plasmid del-CRE. (B) ATRA increased ORMDL3 promoter (pGL3-74) activity, and this increase could be weakened by endogenous CREB knockdown or CREB-binding site deletion. Relative luciferase activity of pGL-74 co-transfected with control siRNA stimulated with DMSO was set as 1. Data represent the mean ± SE of triplicate experiments (*P<0.05 vs. control).

We have previously reported that the core promoter of mouse ORMDL3 gene was located in the region between −74 and+141 relative to TSS (5′ end position of mouse ORMDL3 gene (NM_025661.4) was defined as TSS reference and designated as position+1). CREB plays a pivotal role in regulating ORMDL3 transcription through a CRE element “TGACGTCA”, which locates between −27 and −20 [26]. To determine whether CRE-dependent ORMDL3 transcription could be induced by ATRA, dual-luciferase reporter assays were performed in NIH3T3 cells. We concluded from Fig. 2 that ATRA enhanced ORMDL3 promoter activity via CREB, because ATRA induced 3.5-fold increase of luciferase activity of pGL3-74 while knocking down endogenous CREB by CREB-specific siRNA completely annulated ATRA's induction. Furthermore, CRE element deletion (Fig. 2A) significantly weakened ATRA's induction on ORMDL3 promoter activity. Specifically, ATRA incubation resulted in only 1.8-fold increase in luciferase activity of del-CRE (Fig. 2B). These data suggested ATRA could increase ORMDL3 promoter activity and this increase was achieved partially through the CRE-site in the core promoter.

### CREB Medicated the Up-regulation of ORMDL3 mRNA by ATRA

**Figure 3:**
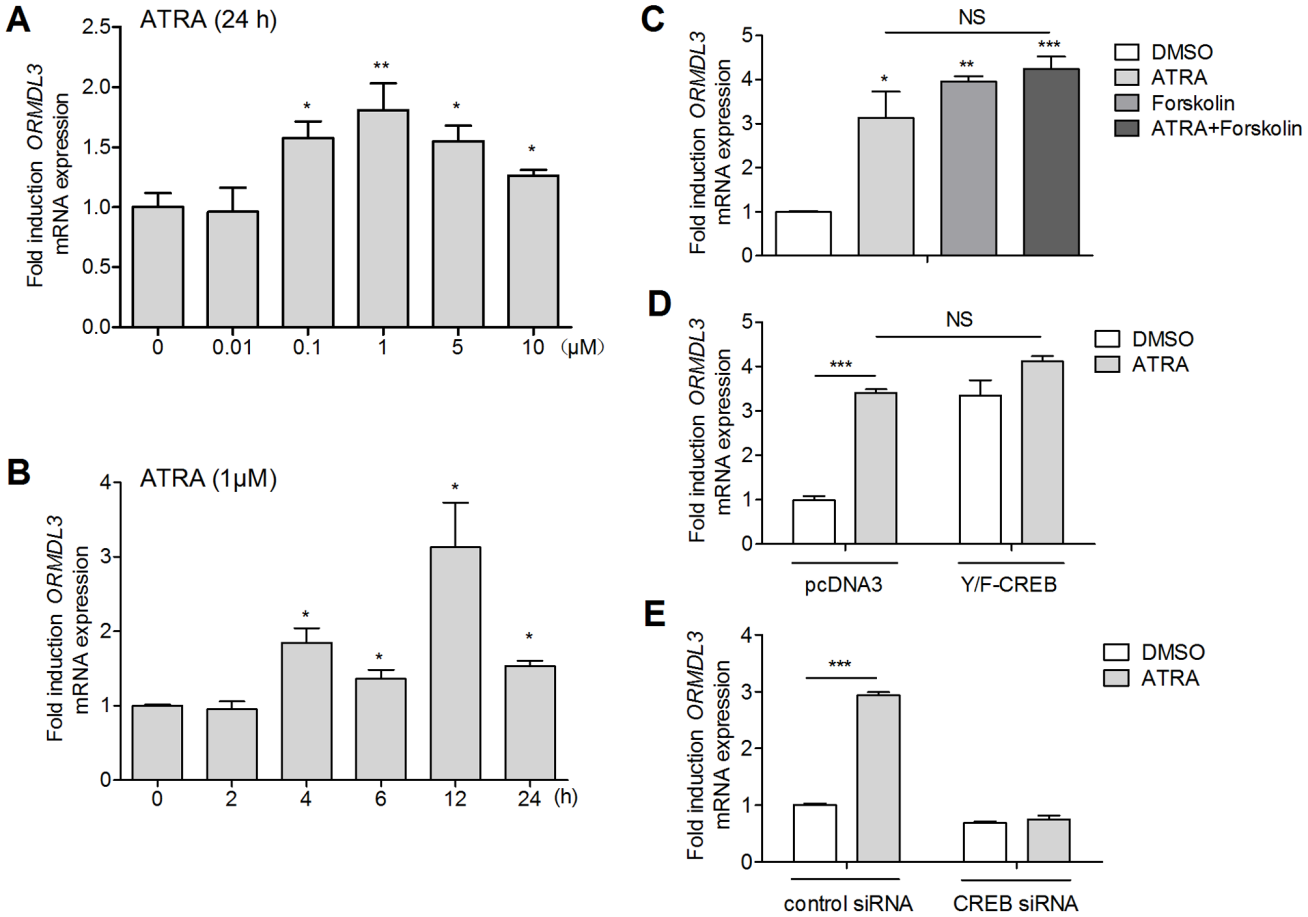
CREB medicated ATRA-induced ORMDL3 mRNA up-regulation in NIH3T3 cells. (A and B) ATRA induced ORMDL3 mRNA transcription in dose- and time- dependent manner. (C) ATRA (1 μΜ) and Forskolin (10 μΜ) incubation alone for 12 h increased ORMDL3 mRNA level, but no synergistic effect was seen when stimulated in combination. (D) CREB overexpression by plasmid Y/F-CREB induced ORMDL3 mRNA transcription, but could not further strengthen ATRA's induction. (E) Knockdown endogenous CREB decreased ORMDL3 mRNA level and abolished ATRA-induced ORMDL3 mRNA production. Data represent the mean ± SE of triplicate experiments (* *P*<0.05; ** *P*<0.01; *** *P*<0.001; NS: not significant).

Effects of ATRA on ORMDL3 mRNA transcription was identified by quantitative real-time PCR. NIH3T3 cells were exposed to various doses of ATRA for 24 h before ORMDL3 mRNA detection. Fig. 3A revealed that ORMDL3 mRNA contents could be significantly increased by ATRA at the concentrations of 0.1–10 μM, and the greatest enhancement was achieved at 1 μM ATRA. Time-course stimulation (Fig. 3B) showed that the ATRA (1 μM) responses of ORMDL3 mRNA was already 1.9-fold after 4 h, 1.2-fold after 6 h, reached a peak of 3.1-fold after 12 h and declined to 1.5-fold after 24 h.

In our previous work, PKA activator, Forskolin, induced ORMDL3 transcription via facilitating CREB phosphorylation in NIH3T3 cells [26]. Here, we found that though ATRA and Forskolin stimulation alone could activate CREB and induce ORMDL3 transcription, there was no significant synergistic effect on ORMDL3 transcription when treated in combination (Fig. 3C). Besides, we applied the CREB expression plasmid, Y/F-CREB, which is a gain-of functional mutant containing a Tyr134Phe mutation that makes CREB behave as a constitutive activator in vivo and stimulates target gene expression like wild type CREB [26]. Agreed with Forskolin, transiently transfecting Y/F-CREB into cells could up-regulate ORMDL3 transcription but could not strengthen ATRA's induction (Fig. 3D). However, knocking down endogenous CREB by siRNA blocked the induction by ATRA (Fig. 3E).

### The Cross-talk between ATRA and PKA/CREB Signaling on ORMDL3 Regulation

**Figure 4:**
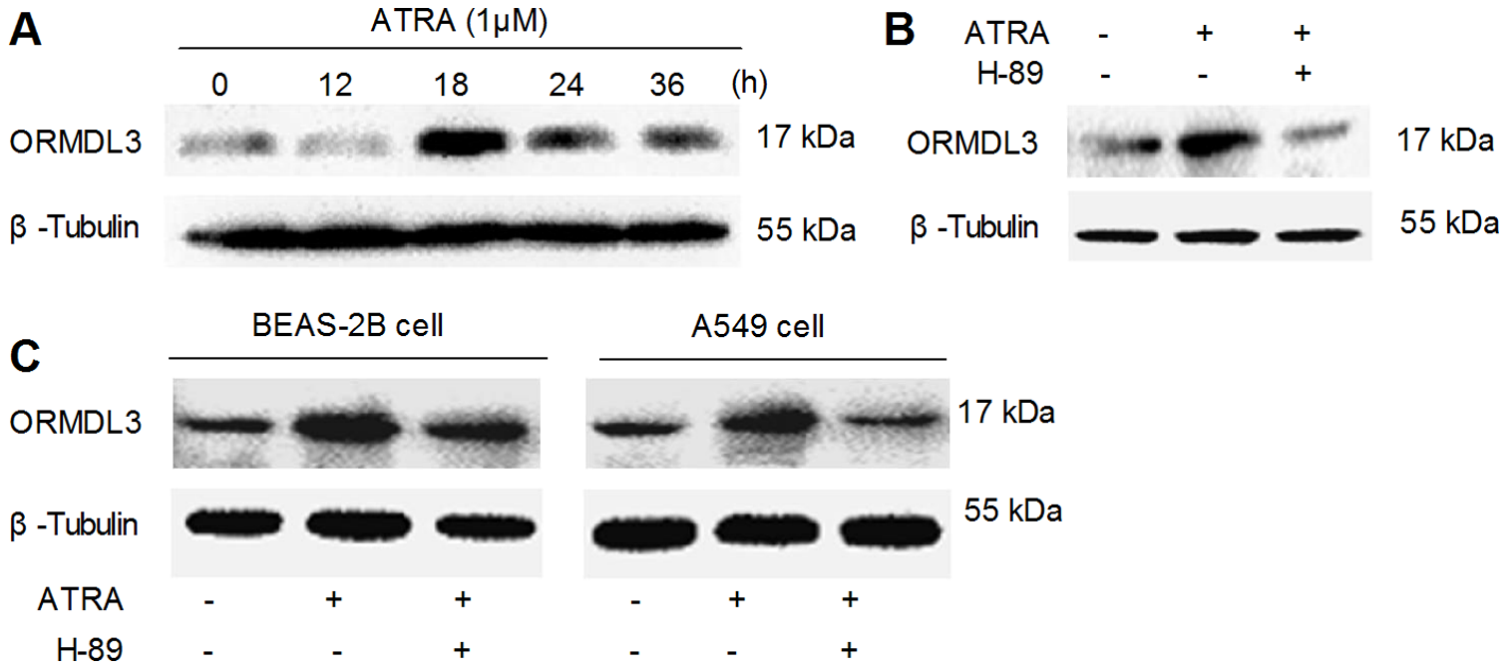
ATRA-induced ORMDL3 production depended on PKA. (A) ATRA induced ORMDL3 protein expression in a time-dependent manner in NIH3T3 cells. (B) Pre-treatment with H-89 suppressed ATRA-induced ORMDL3 production of NIH3T3 cells, BEAS-2B cells and A549 cells.

PKA is the major kinase that activates CREB in vivo and cAMP/PKA pathway is an integrated part of ATRA signal network, this stimulated us to investigate the possible role of PKA pathway in ATRA-induced ORMDL3 expression. Fig. 1B have showed that a 60-minute pre-incubation with PKA inhibitor, H-89 (15 μM) could block ATRA-induced CREB activation, suggesting that PKA might play an important mediating role in ATRA-induced ORMDL3 expression. In Fig. 4A, we presented that ATRA increased ORMDL3 protein level in a time-dependent manner in NIH3T3 cells. In detail, the increase peaked up 18 h after ATRA treatment, followed by a slight decrease between 24 h and 36 h, but still higher than untreated control group. Importantly, a 60-min pre-incubation with H-89 (15 μM) completely inhibited ATRA-induced ORMDL3 production (Fig. 4B). Furthermore, PKA-dependent ORMDL3 induction by ATRA was also observed in human bronchial epithelial cells BEAS-2B and human type II alveolar lung epithelium cells A549 (Fig. 4C).

### Differential Effects of RAR-α Agonist and Retinoic Acid Isoforms on ORMDL3 Modulation

**Figure 5:**
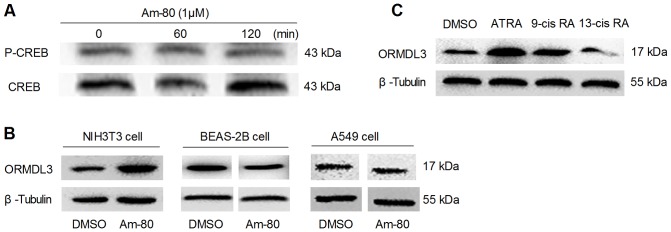
Different effects of AM-80, 9-cis RA and 13-cis RA on ORMDL3 expression. (A) NIH3T3 cells incubated with Am-80 (1 μΜ) for 60-min or 120-min had no significant impact on CREB phosphorylation. (B) ORMDL3 protein of NIH3T3 cells was increased after incubated with Am-80 for 18 h; production of ORMDL3 in BEAS-2B and A549 cells were not affected by Am-80. (C) 9-cis RA (1 μΜ) up-regulated and 13-cis RA (1 μΜ) down-regulated ORMDL3 expression in NIH3T3 cells after 18 h incubation.

RAR/RXR signaling is the traditional pathway that mediates biological effects of retinoic acid (RA) through binding to the retinoic acid response elements (RAREs) in regulatory regions of target genes, and the α-receptor has been found to be most important. ATRA, 9-cis retinoic acid (9-cis RA) and 13-cis retinoic acid (13-cis RA) all bind to RAR-α, but only 9-cis RA binds to and activates RXR-α. We employed an RAR-α agonist, Am 80, to examine whether RAR/RXR signaling is involved in ORMDL3 modulation. The online software TFSEARCH ver.1.3. analysis haven't identified any RARE in the potential promoter region (up to 2000 bp 5′ of TSS), introns and 3′-downstream regions of mouse and human ORMDL3 gene with threshold score: 85.0. However, Am-80 stimulation for 18 h induced 2.1-fold ORMDL3 expression without the accompany of CREB phosphorylation in NIH3T3 cells (Fig. 5). Nevertheless, in BEAS-2B and A549 cells, Am-80 incubation did not show any impact on ORMDL3 production. Semi-quantitative RT-PCR analysis indicated that the expression of RAR-αmRNA in NIH3T3 cells was 8.4-fold that of BEAS-2B cells and 3.9-fold that of A549 cells (data were not shown here). Thus, the different responses of these cell lines to Am-80 might be due to the different RAR-αexpression patterns.

In addition, we also examined the effects of 9-cis RA and 13-cis RA on CREB activation and ORMDL3 expression in NIH3T3 cells. Incubation with 9-cis RA and 13-cis RA had no influence on CREB phosphorylation (data were not shown here), but stimulation with 9-cis RA (1 μM) for 18 h up-regulated ORMDL3 protein level by 1.8-fold, whereas 13-cis RA (1 μM) resulted in a 60% decrease in ORMDL3 production (Fig. 5C). These data suggested that RAR/RXR pathway might not directly regulate ORMDL3 expression, but rather influencing ORMDL3 expression indirectly, and these influences might be cell specifically and differential upstream signals might result in differential outcomes.

## Discussion

RA normally activates gene expression through binding to nuclear receptors that interact with RAREs in regulatory regions of target genes. However, RA has extra-genic effects and activates signaling molecules depending on cell type [30]. Zhao et al. reported that ATRA induces rapid elevation of intracellular cAMP level and activation of PKA to promote acute promyelocytic leukemia cells differentiation [27]. In neuronal cells, ATRA induces ERK1/2 phosphorylation, which in turn activates CREB and promotes CREB-dependent transactivation [31]. Aggarwal et al. showed that RA rapidly activates CREB via PKC, ERK1/2, and RSK in a retinoid receptor-independent manner in normal bronchial epithelial cells [32]. Here, we showed that ATRA-induced phosphorylation of CREB leads to a direct stimulation on ORMDL3 transcription in NIH3T3 cells, and this stimulation is dependent on PKA since omitting PKA by using PKA-specific inhibitor completely abolished ATRA-induced CREB activation and ORMDL3 production. Besides, CRE element in ORMDL3 promoter share high homology between mouse and human [26] might explain why ATRA-induced ORMDL3 production also could be observed in human cells BEAS-2B and A549. However, here we cannot know whether other CREB activators, such as PKC, ERK1/2 and RSK are involved in ATRA-mediated CREB activation.

RAR/RXR-RARE signaling is the traditional pathway via which RA modulating target genes expression. The RARs/RXRs heterodimers normally bind to RARE in the absence of ligands, repressing transcription by recruiting nuclear receptor corepressor complexes that contain histone deacetylases. ATRA at physiologic concentrations triggers the dissociation of corepressors and the association of coactivators with histone acetyltransferase activities. This process leads to activation of ATRA-responsive genes via alteration of chromatin structure and enables cellular differentiation of normal myelomonocytic progenitor cells [33]. Compared with the endogenously formed isoforms ATRA and 9-cis RA, 13-cis RA has relatively weak transactivation activity for RAR/RXR, and it may possess a novel signaling pathway of its own [34]. It has reported that ATRA could be, at least partially, converted into 9-cis RA and 13-cis RA in vitro, but 9-cis RA and 13-cis RA may produce biological effects distinct from those produced by ATRA [35]. In our study, bioinformatic analysis did not identify any RARE in regulatory region of mouse and human ORMDL3, but ORMDL3 did show different responses to RAR-α agonist, 9-cis RA and 13-cis RA. In addition, the inducing effect of ATRA on CREB phosphorylation could not be mimicked by RAR-α agonist, 9-cis RA or 13-cis RA. These results further confirmed that ATRA can modulate target gene by a nongenomic mechanism, and 9-cis RA and 13-cis RA may have their own specific signaling. Studies have shown that 13-cis RA can act as a potent inhibitor of many of the retinoid and hydroxysteroid mediated pathways [36]. A recent research showed that 13-cis RA mediated activation of ERK1/2, is capable of disrupting transcription factor Sp1, the requisite transactivator for angiotensin type 1A receptor (AT1AR) gene transcription, leading to an inhibition on AT1AR expression [34]. Besides, in our study, we only focused on transcription factor CREB, we could not rule out whether RA or RAR may in conjunction with other transcription factors such as SP1/3 and modulate ORMDL3 expression. Further extensive studies are needed to answer the question of what 13-cis RA target molecule initiates the inhibitory effect on ORMDL3 expression since it is of potential beneficial action on asthma.

Some clinical studies have demonstrated that dietary intake of Vitamin A is associated with the occurrence of asthma, Vitamin A intake is significantly lower in people with asthma than in those without asthma and in people with severe asthma than in those with mild asthma [37]. Vitamin A may be of value on asthma control. In vitro mechanistic studies and animal model studies supported this hypothesis as Vitamin A or its active form ATRA: protects airway from oxidative damage and maintains airway epithelial integrity [38]; up-regulates transforming growth factor-β, matrix metalloproteinase 9, hepatocyte growth factor receptor and β1-integin expression, and promotes airway epithelial repair [15]; facilitates Treg cells differentiation and induces immune tolerance [39], [40]. However, Checkley et al. reported [41] that Vitamin A supplementation early in life is not associated with a decreased risk of asthma in an area with chronic Vitamin A deficiency. Moreover, some other studies showed that early life supplementation with Vitamin A is associated with increased risk for asthma [42], [43], and for adults cod liver oil intake increases incidence of asthma [44]. Some researchers thought that Vitamin A promotes Th2 bias may exacerbate allergic immune and inflammatory responses. Both in vivo and in vitro studies confirmed that Vitamin A and ATRA induce Th2 bias and promote Th2 cytokines realise, such as IL-4, IL-5, IL-13 and GATA-3 [45], [46], [47], which is one of the major characteristics of asthma. Thus, application of Vitamin A on asthma treatment is still controversial. Our data presented a new potential side effect of Vitamin A application on asthma control because the major metabolite of Vitamin A, ATRA, promoted ORMDL3 production in both mouse and human cell lines, and this may become a starting point for more detailed mechanism researches on ATRA and asthma.

Miller et al. reported that [9] allergen could significantly induce ORMDL3 mRNA expression in A549 cells and primary bronchial epithelial cells in vitro; Th2 cytokines IL-4 and IL-13 induce expression of ORMDL3 mRNA in bronchial epithelium in vivo; overexpress of ORMDL3 in primary normal human bronchial epithelial cells rusulted in high levels of MMP-9, CC chemokines (CCL-20), CXC chemokines (IL-8, CXCL-10, CXCL-11), and oligoadenylate synthetases (OAS) genes. We studied the influence of ATRA on ORMDL3 expression in vitro, but whether ATRA-induced Th2 cytokines in vive would affect ORMDL3 production is unknown. In addition, RA plays pivotal roles in promoting CD4^+^T cells differentation and affecting T cells polarization direction [48]. It has reported that ORMDL3 negatively modulates Ca^2+^ release-activated Ca^2+^ currents (I (CRAC)), store-operated calcium entry (SOCE), nuclear factor of activated T cell nuclear translocation and IL-2 production, which in turn influence T cell activation [12]. Our data suggested that ORMDL3 may be a signal molecule that mediates ATRA-regulated T cell activation.

In summary, we demonstrated here that ATRA facilitates ORMDL3 production in mouse embryonic fibroblast cells NIH3T3, human bronchial epithelial cells BEAS-2B and human type II alveolar lung epithelium cells A549 via inducing CREB phosphorylation which in turn promotes ORMDL3 transcription. Besides, though there was no retinoic acid response element in regulatory region of ORMDL3, the involvement of RAR/RXR pathway in ORMDL3 regulation could not be excluded since ORMDL3 showed different responses to RAR-α agonist (Am-80) and RAR/RXR upstream signal molecules (9-cis RA and 13-cis RA) without the accompany of increased CREB phosphorylation. Anyhow, the facilitation of ATRA on ORMDL3 production should be taken in to account since this may become another side effect of Vitamin A application on asthma treatment.
